# Molecular Heterogeneity of Ewing Sarcoma as Detected by Ion Torrent Sequencing

**DOI:** 10.1371/journal.pone.0153546

**Published:** 2016-04-14

**Authors:** Nana Zhang, Haijing Liu, Guanjun Yue, Yan Zhang, Jiangfeng You, Hua Wang

**Affiliations:** 1 Department of Pathology, School of Basic Medical Sciences, Peking University Health Science Center, Beijing, China; 2 Department of Pathology, Peking University Third Hospital, Beijing, China; CNR, ITALY

## Abstract

Ewing sarcoma (ES) is the second most common malignant bone and soft tissue tumor in children and adolescents. Despite advances in comprehensive treatment, patients with ES metastases still suffer poor outcomes, thus, emphasizing the need for detailed genetic profiles of ES patients to identify suitable molecular biomarkers for improved prognosis and development of effective and targeted therapies. In this study, the next generation sequencing Ion AmpliSeq^™^ Cancer Hotspot Panel v2 was used to identify cancer-related gene mutations in the tissue samples from 20 ES patients. This platform targeted 207 amplicons of 2800 loci in 50 cancer-related genes. Among the 20 tissue specimens, 62 nonsynonymous hotspot mutations were identified in 26 cancer-related genes, revealing the molecular heterogeneity of ES. Among these, five novel mutations in cancer-related genes (*KDR*, *STK11*, *MLH1*, *KRAS*, and *PTPN11*) were detected in ES, and these mutations were confirmed with traditional Sanger sequencing. ES patients with *KDR*, *STK11*, and *MLH1* mutations had higher Ki-67 proliferation indices than the ES patients lacking such mutations. Notably, more than half of the ES patients harbored one or two possible ‘druggable’ mutations that have been previously linked to a clinical cancer treatment option. Our results provided the foundation to not only elucidate possible mechanisms involved in ES pathogenesis but also indicated the utility of Ion Torrent sequencing as a sensitive and cost-effective tool to screen key oncogenes and tumor suppressors in order to develop personalized therapy for ES patients.

## Introduction

Ewing sarcoma (ES) is an aggressive and poorly differentiated tumor of the bone or soft tissues. It preferentially occurs in children and young adults, with a peak occurrence at an age of 15 years [1]. With current multidisciplinary therapy, the five-year survival rate for localized disease is approximately 70%, but the overall survival of patients with metastatic or relapsed disease is lower than 30% [2,3]. Few drugs, including intensified chemotherapeutics, are available for the treatment of ES patients with metastatic disease. Moreover, current chemotherapeutic agents are sometimes associated with side effects, which may impart risk to the patients. In-depth explorations of the precise mechanism of ES tumorigenesis and relevant molecular biomarkers for improved prognosis are urgently warranted. Molecularly, ES is characterized by highly recurrent translocations involving ETS transcription factors, with EWS-FLI1 translocations being the most common and found in almost 85% of the patients [1]. Although the role of *EWS-ETS* oncogenes in ES tumorigenesis and progression has been extensively studied, there is a lack of effective therapy that directly targets these transcriptional dynamics. Furthermore, studies highlighting the recurrent frequencies of, albeit few, somatic mutations in *TP53* (5%-20%), *STAG2* genes (20%) and homozygous deletions of *CDKN2A* (10%-30%) have been described in ES pathogenesis [4–6]. Despite such information, the landscape of ES molecular oncogenesis is not completely known. Detailed profiling of the genetic aberrations in ES will improve our understanding of this type of tumor and aid in prognostication. Comprehensive genetic information is also valuable for suitable personalized therapy, which can help to maximize therapeutic efficiency and perhaps even minimize risks associated with chemotherapy.

In recent years, next-generation sequencing (NGS) technologies have allowed deep sequencing of hundreds of genes, thereby facilitating opportunities to identify clinically relevant cancer mutations in order to improve prognosis, diagnosis, and treatment. In addition, Ion Torrent NGS has substantial advantages over conventional sequencing primarily because of its high multiplexing capacity, drastically decreased turnaround time and cost, and limited need of starting material. In this study, we used the Ion AmpliSeq^™^ Cancer Hotspot Panel v2 on the Ion Torrent Personal Genome Machine (IT-PGM) to assess the variety of tumor-associated changes, even with low allelic frequency, in formalin-fixed, paraffin-embedded (FFPE) specimens from 20 ES patients. The panel used targeted 207 amplicons encompassing 2800 known cancer-relevant variants across 50 cancer-related genes. In this study, we systematically analyzed multiple genomic variants found in our ES cohort in order to understand the mechanisms of ES malignant progression and discover potential therapeutic targets and prognostic biomarkers for ES.

## Materials and Methods

### Clinical specimens

The study was approved by the Human Research Ethics Committee of Peking University, Beijing, China. 20 archived FFPE ES specimens were obtained from the Department of Pathology in Peking University Third Hospital, and the institutional ethics committee waived the need for informed consent. Patient information, including sex, age, site, state of metastasis, and prognosis were recorded. All samples and medical data used in the study were anonymized. Tumor samples were all obtained from the primary disease site and were not exposed to chemotherapy or radiotherapy before the surgery.

### Immunohistochemistry

Immunohistochemical assay for CD99, FLI1, synaptophysin (Syn), chromogranin (CgA), neuron-specific enolase (NSE), S-100, and Ki-67 proteins (S1 Table) were performed on 4-μm-thick sections from FFPE tissue by EnVision two-step immunostaining. Deparaffinized slides were rinsed, then sequentially incubated with the primary antibody and the secondary biotinylated antibody (Dako, Glostrup, Denmark). The staining was visualized after incubation with 3,3′-diaminobenzidine (DAB) chromogen. Tissue samples that were not treated with specific primary antibodies were used as blank controls. Ki-67 index score was defined by the percentage of tumor cells with positive nuclear staining.

### Fluorescence in Situ Hybridization (FISH)

FISH evaluation for *EWSR1* rearrangement was performed on 3 μm-thick sections of the 20 FFPE ES samples using the LSI *EWSR1* (22q12) Dual Color, Break Apart Rearrangement Probe (Vysis, Abbott Molecular, Des Plaines, IL, USA). Nuclei were counterstained with 4',6-diamidino-2-phenylindole (DAPI). In intact nuclei, split red and green signals indicated the presence of a *EWS* (22q12) gene rearrangement, while fused signals indicated intact 22q12 alleles of the chromosome. At least 200 nuclei per case were evaluated via fluorescence microscopy (Olympus, Tokyo, Japan). A positive rearrangement of *EWSR1* was defined as more than 10% of tumor cells having split signals.

### DNA extraction

Hematoxylin and Eosin (H&E) stained tissue sections were reviewed by pathologists who marked tumorous areas and indicated the tumor percentage. Only specimens with >80% tumor cells in the marked area were included in the study. DNA was extracted from five to eight unstained, 10 μm-thick tissue sections of FFPE ES specimens using the QIAamp DNA FFPE Tissue Kit (Qiagen, Hilden, Germany), according to the manufacturer’s instructions. Quantity of DNA was determined by a Qubit 2.0 Fluorometer (Life Technologies, Carlsbad, CA, USA).

### Construction of Targeted Amplicon Libraries and NGS Sequencing

A total of 20 ES specimens were subjected to NGS on the IT-PGM platform. Libraries were prepared using as low as 10 ng of input DNA with the Ion AmpliSeq^™^ Library Kits 2.0 (Life Technologies, Carlsbad, CA, USA), following the manufacturer’s instructions. Genomic DNA amplification was carried out using the Ion AmpliSeq^™^ Cancer Hotspot Panel v2 and 5x Ion AmpliSeq^™^ HiFi Master Mix (Life Technologies, Carlsbad, CA, USA). The panel was designed to detect 2800 hotspots of 50 oncogenes and tumor suppressor genes. Each sample was individually barcoded with the Ion Xpress Barcode Adapters Kit (Life Technologies, Carlsbad, CA, USA). The adapters-ligated amplicons were purified with AMPure beads (Beckman Coulter, Brea, CA, USA). After a second round of amplification for 20 cycles, the AMPure beads were used for the final purification step. Quantity of DNA amplicons was evaluated using Qubit 2.0 Fluorometer.

The barcoded DNA library absorbed on the Ion Sphere^™^ Particles (ISPs) was subjected to emulsion PCR using an Ion OneTouch^™^ 200 Template Kit v2 DL (Life Technologies, Carlsbad, CA, USA). The subsequent isolation of ISPs with DNA was performed with the Ion OneTouch^™^ ES (Life Technologies, Carlsbad, CA, USA). Next, the enriched template-positive ISPs were loaded on a 316 Chip according to the Ion PGM^™^ 200 Sequencing protocol.

### Data analysis

Sequencing reads generated were analyzed using the Intuitive Torrent Suite software program with the ‘variant caller v4.0.2’ plugin. Gene alterations were finally collected into a standardized Variant Call Format (VCF) and aligned to the human reference genome, hg19, which was uploaded on the Ion Reporter software v4.2 to perform variant calling and mapping. In order to minimize the risk for potential errors in base calling, several filtering steps were followed (S1 Fig): (1) the average depth of total coverage was >100, each variant coverage was >20, variant frequency of each specimen was >5%, and quality score (QC) was >20, corresponding to 1 base error allowed per 100 bases; (2) mutations were examined using Integrative Genomics Viewer software (http://www.broadinstitute.org/igv) to eliminate sequencing errors or strand bias; and (3) amplicon AMPL339432 (*PIK3CA*, exon13, chr3:178938822–178938906) was eliminated, because it did not uniquely matched with the human genome or it exhibited false positive frequency.

Next, the detected mutations were compared with variants present in the Catalogue of Somatic Mutations in Cancer database (COSMIC, http://www.cancer.sanger.ac.uk/cosmic/) [7] and database of Single Nucleotide Polymorphisms (dbSNP) to distinguish known somatic mutations from germline mutations. Software tools SIFT (v4.0.3) and PolyPhen-2 (v2.1.0) were also applied to predict whether an amino acid substitution affected protein function. SIFT scores ranged from 0.0 (deleterious) to 1.0 (tolerated). Variants with scores in the 0.0–0.05 range were considered deleterious. Scores closer to 0.0 were more confidently predicted to be deleterious. On the contrary, variants with PolyPhen-2 scores in the 0.0–0.15 range were considered to be benign, and scores in the 0.15–1.0 range were considered possibly damaging. Scores in the 0.85–1.0 range were more confidently predicted to be damaging. Furthermore, the ClinVar database (http://www.ncbi.nlm.gov/clinvar/); the International Cancer Genome Consortium (ICGC) Data Portal, a web tool for exploring and analyzing multiple cancer genomics data [8]; and previous reports were used to explore variants with potential clinical value during our data analysis.

### Sanger sequencing

Some detected variants were confirmed by Sanger sequencing. Due to limited sensitivity of Sanger sequencing as compared to Ion Torrent NGS, we selected mutated sites in which variant allele frequencies were >15% for the validation.

## Results

### Patient characteristics

**Fig 1:**
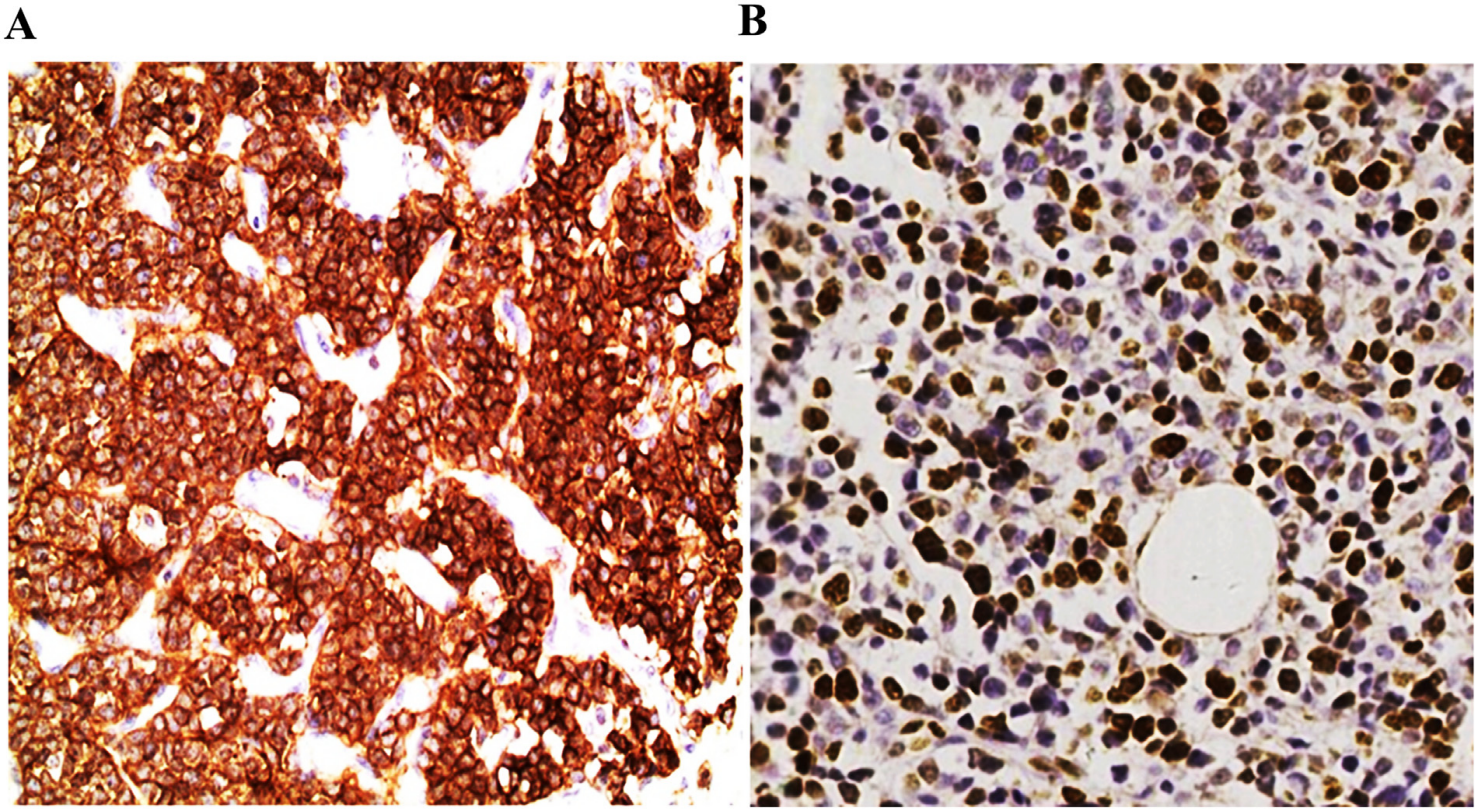
Immunohistochemical staining of ES for CD99 and Ki-67. A) CD99 staining, with strong membrane positivity (400×) and B) Ki-67 staining, with strong nuclear positivity (400×).

**Fig 2:**
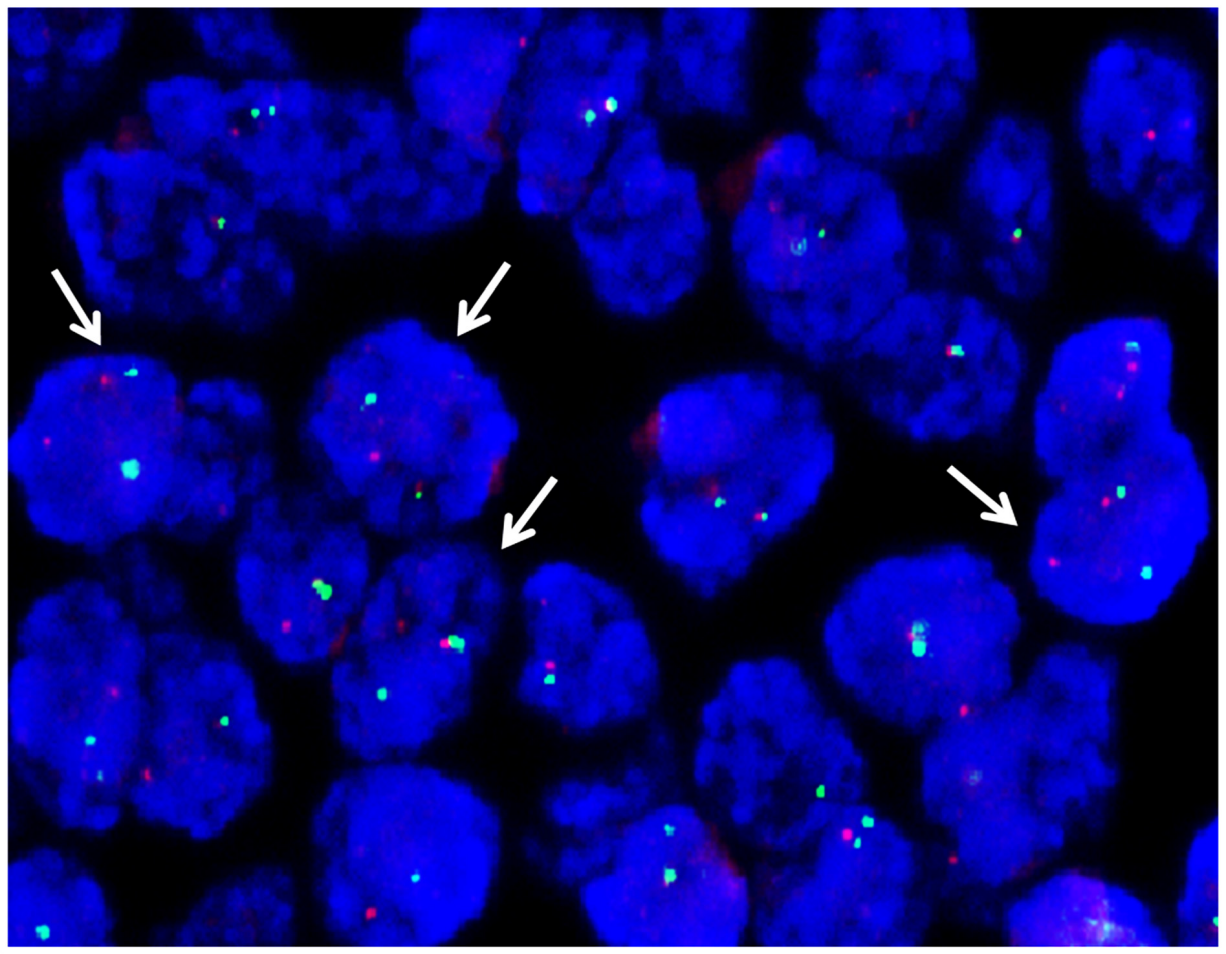
Fluorescence in situ hybridization for the assessment of *EWSR1* gene rearrangements in ES samples. Nuclei of tumor cells with *EWSR1* rearrangements were detected as red-green split signals as represented by the arrow.

A total of 20 ES patients were involved in the current study (Table 1). The median age was 15.5 years old (range, 2–70 years). The positive staining of CD99 was diffusely observed in all the cases. Ki-67 proliferation index was from 3%-80% in the 20 specimens (Fig 1). Moreover, FISH evaluation using break-apart rearrangement probes for the *EWSR1* gene on 22q12 showed *EWSR1* gene rearrangements in the whole cohort (Fig 2). Thus, both CD99 and FISH assays supported the diagnostic basis for ES.

**Table 1:** Patient information for 20 ES cases.

Samples	Sex	Age	Tumor site	CD99	*EWSR1* gene rearrangements	Survival(months)
T1	M	6	Head	**+**	detected	7
T2	F	22	Chest wall	+	detected	Alive
T3	F	27	Kidney	**+**	detected	2
T4	M	21	Cervical vertebra	**+**	detected	4
T5	M	9	Chest wall	**+**	detected	19
T6	M	70	Pleura	**+**	detected	10
T7	F	24	Kidney	**+**	detected	38
T8	M	47	Retroperitoneum	**+**	detected	1
T9	M	10	Cervical vertebra	**+**	detected	Alive
T10	M	27	Thoracic vertebra	**+**	detected	Alive
T11	M	12	Cervical vertebra	**+**	detected	Alive
T12	F	39	Ankle	**+**	detected	NA
T13	F	16	Pelvis&Pubis	**+**	detected	Alive
T14	F	15	Femur	**+**	detected	NA
T15	M	2	Scrotum	**+**	detected	NA
T16	F	12	Femur	**+**	detected	NA
T17	M	15	Scapula	**+**	detected	NA
T18	M	37	Thoracic vertebra	**+**	detected	17
T19	M	15	Upper arm	**+**	detected	NA
T20	M	12	Chest wall	**+**	detected	23

Abbreviations: M, male; F, female; NA, not available.

### Genetic landscape of variants on the IT-PGM platform

The Ion AmpliSeq^™^ Cancer Hotspot Panel v2 targeted 207 amplicons covering “mutation hotspots region” in 50 tumor-related susceptibility genes and protective genes (S2 Table). Among the 20 ES specimens, a total of 62 nonsynonymous coding variants of 26 genes were found in the cancer-specific COSMIC database (S3 Table). Alterations of these hot-spots genes consisted of 87.1% missense mutations (54/62) and 12.9% nonsense mutations (8/62).

**Fig 3:**
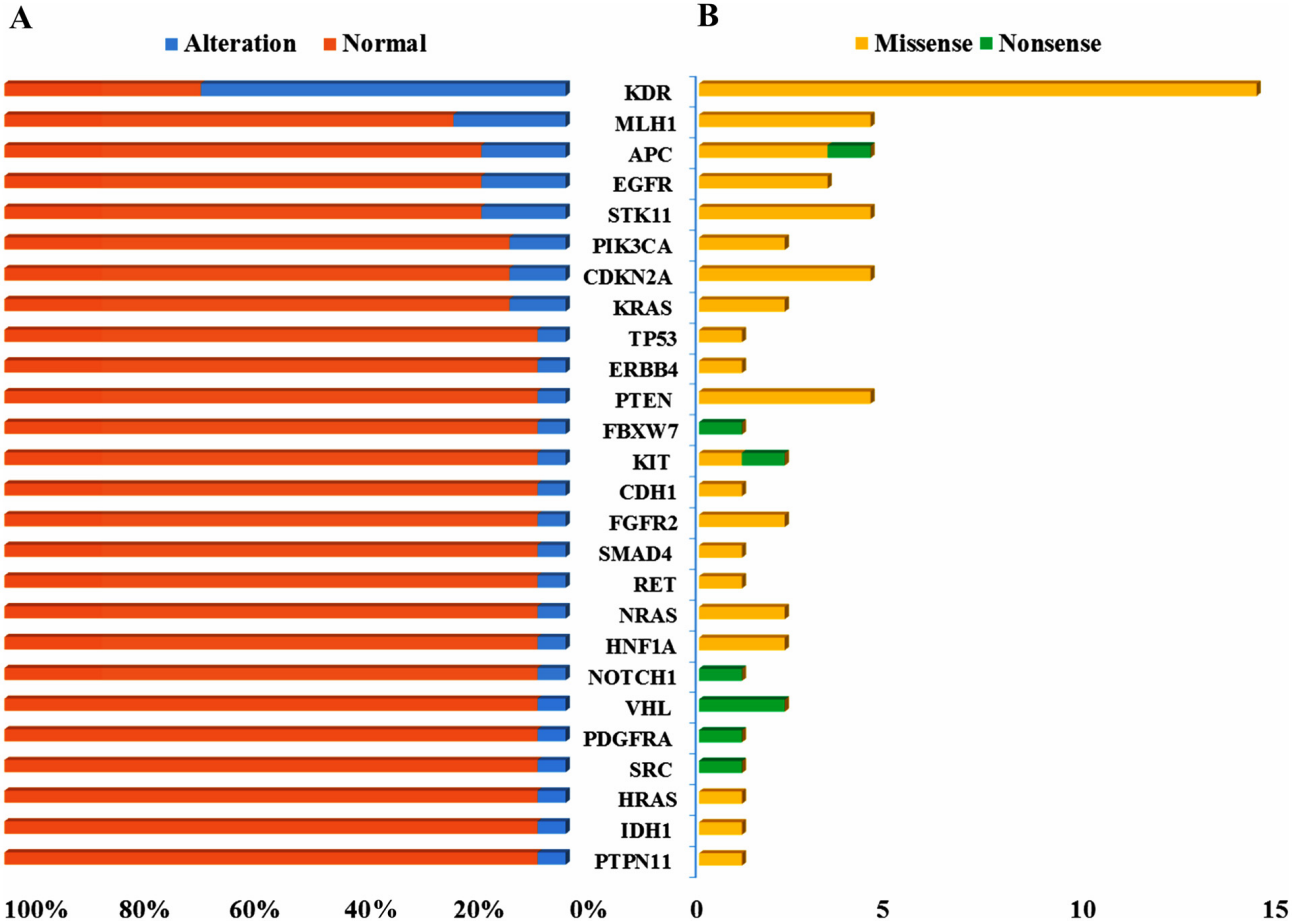
Overall alteration distribution of 26 genes annotated by the COSMIC database. A) Mutation frequency of altered genes in 20 ES samples. The blue bars represent alterations in the ES samples, whereas the red bars represent ES samples without alterations. B) Mutation types of the 26 genes. Yellow bars represent missense mutations, and green bars represent nonsense mutations.

Among the 26 genes, eight genes, *KDR*, *MLH1*, *APC*, *EGFR*, *STK11*, *PIK3CA*, *CDKN2A*, and *KRAS*, were found mutated in at least two ES samples (Fig 3). In addition, about 35% (7/20) of ES cases had at least two hotspot mutated genes in our cohort as assessed by IT-PGM. Alterations of some genes were verified by Sanger sequencing (S4 Table). In multiple ES patient samples, *KDR* mutation was found at the same site (n = 12), as well as mutations in *MLH1* (n = 4), and *STK11* (n = 3) at separate sites. ES with these mutations correlated with a higher Ki-67 proliferation index. For *KDR* mutation, the Ki-67 indices were above 10% in all patients except one, and in cases with *MLH1* mutation, the score was no less than 10%. For *STK11* mutation, the Ki-67 index varied from 40% to 50%.

### Mutation hotspots and Novel mutations in ES

Referring to the ClinVar, ICGC, COSMIC databases and relevant literatures, some common hotspot mutated sites in 12 genes were observed by IT-PGM. Particularly, *KDR*, *STK11*, *MLH1*, *KRAS*, *PIK3CA*, *APC*, *EGFR*, *FGFR2*, *HNF1A*, *VHL*, *IDH1*, and *PTPN11* were reported to have potential detrimental effects on the patients, some of which were predicted by the SIFT and PolyPhen-2 software. The distribution of these gene mutations are summarized in Table 2. Most of the hotspot mutations, such as *KRAS*, *PIK3CA*, *EGFR*, *FGFR2*, *HNF1A*, *VHL*, *IDH1* and *PTPN11*, were found mutated in a variety of tumors [9–11]. Variants in these genes were also detected in ES. However, there were some differences in the location of the individual variants in our study.

**Table 2:** Deleterious mutated genes identified in ES samples by IT-PGM.

Gene	Exon	cDNA change	Protein change	Mutation type	COSMIC ID	ClinVar	ICGC	SIFT	PolyPhen-2
*KDR*	11	c.1416A>T	p.Q472H	missense	COSM149673	---	low	---	---
*STK11*	8	c.1062C>G	p.F354L	missense	COSM21360	pathogenic	---	0.20	0.03
*KRAS*	2	c.35G>A	p.G12D	missense	COSM521	pathogenic	low	0.00	0.80
*MLH1*	12	c.1151T>A	p.V384D	missense	COSM26085	benign	---	0.00	1.00
*PIK3CA*	2	c.323G>A	p.R108H	missense	COSM27497	---	low	0.01	1.00
*PIK3CA*	10	c.1612G>A	p.D538N	missense	COSM21467	---	low	0.01	1.00
*APC*	16	c.3875C>T	p.T1292M	missense	COSM1432296	uncertain	high	0.01	0.35
*EGFR*	3	c.340G>A	p.E114K	missense	COSM174732	---	low	0.05	0.02
*FGFR2*	7	c.758C>T	p.P253L	missense	COSM537803	---	high	---	---
*HNF1A*	4	c.815G>A	p.R272H	missense	COSM24933	pathogenic	---	0.00	1.00
*VHL*	3	c.481C>T	p.R161*	missense	COSM17612	pathogenic	high	---	---
*IDH1*	4	c.394C>T	p.R132C	missense	COSM28747	---	high	0.00	0.26
*PTPN11*	3	c.215C>T	p.A72V	missense	COSM13015	---	high	0.00	0.99

‘---’ refers to no predictions by the database and software. The ‘low’ or ‘high’ annotation by ICGC means the potential effect to patients who have the mutation. SIFT scores ranged from 0.0 (deleterious) to 1.0 (tolerated). Variants with scores 0.0–0.05 were considered deleterious, and scores closer to 0.0 were more confidently predicted to be deleterious. On the contrary, variants with PolyPhen-2 scores ranged from 0.0 to 0.15 were predicted to be benign, and scores 0.15 to 1.0 were considered possibly damaging. Additionally, scores from 0.85 to 1.0 were more confidently predicted to be damaging.

‘*’refers to nonsense mutation.

For *KDR*, two novel mutated sites were detected in ES. One was the Q472H mutation (c.1416A>T) in exon 11 and was found in 12 ES specimens, which were all confirmed by Sanger sequencing (S4 Table). This mutation is located in the fifth NH2-terminal Ig-like domains, playing an important role in ligand binding and was reported in the colorectal liver metastasis samples from a colorectal carcinoma cohort [12,13]. The other *KDR* gene mutation was R962H, caused by G>A transition, in a ES sample and is involved in the catalytic domain of tyrosine kinase. This mutation was also discovered in a leukemia cell line [14].

In the study, two novel *STK11* mutations were found in 3 ES patients. A C>G transversion at chr19:1223125, led to a missense mutation (F354L) and was located near tyrosine kinase domain of STK11. As per the ICGC records, breast, large intestine, and lung cancer patients possess this mutation, but its oncogenic influence is unknown. One of the three cases also harbored another *STK11* hotspot (D350N) mutation induced by a G>A transition.

For *KRAS*, a total of two missense mutations were separately found in two ES cases, both along exon 2. Among these mutations, an amino acid substitution, G12D, resulting from the c.35G>A transition was found in other tumor types and annotated as pathogenic to the patient prognosis [9]. The other alteration, G13S, was due to c.37G>A and rarely occurred in tumors [15]. Notably, both the variants were located along the GTP binding domain of KRAS. Mutations in this conserved domain most likely activate KRAS signaling pathways, thus influencing its respective downstream signaling cascade.

Other novel gene mutations detected from ES in the study were in the *MLH1* and *PTPN11* genes. V384D in *MLH1* resulted from transversion mutation c.1151T>A. This mutation discovered in four ES patients, and the same mutation site was reported in epithelial ovarian cancer [16]. The A72V amino acid in *PTPN11* induced by c.215C>T transition was found in one ES specimen. This mutation is located in the SH2 domain.

In three ES samples, each patient had a mutated site in *EGFR*. The amino acid change E114K of the L-Domain resulted from the c.340G>A mutation on exon 3. It has pathogenic potential for ES patients according to the ICGC. The other two amino acid substitutions, E746K on exon 19 and G810S on exon 20, were in the tyrosine kinase domain, thus leading to the activation of downstream pathways involved in cell differentiation, proliferation, and survival [15,17].

Alterations in *HNF1A* lead to R272H amino acid change, and R161* change was found in the alpha domain of VHL. Both mutations were most likely pathogenic. A C>G transversion induced variants in IDH1 (R132C amino acid change) along the dehydrogenase-like domain was reported in other types of tumors, and possibly responsible for a high function impact [18].

Four kinds of *CDKN2A* mutations were also detected in two ES patients. In exon 2, amino acid changes in R99Q, N71D, and A57T, located in the ankyrin repeats domain, appeared in one ES case. The other mutation, V115L, was also located in the same functional domain. Though isopropylmalate the same alteration of the above genes were seen in other types of tumors, the significance of which with regards to ES is novel.

In *APC*, four variants (T1292M, P1369S, V1352I, and Q1123*) occurred in three ES samples. The first two, located in cysteine rich repeat domain, were all caused by C>T transition in the same patient. Another patient had a V1352I mutation induced by c.4054G>A, which was previously discovered in invasive urothelial carcinomas [19]. The three variants above were located in regions adjacent to 20 amino-acid repeats binding sites for β-catenin [20].

Among the 50 tumor-related genes in our study, about half were druggable target genes or related to signaling pathways (Table 3). Among which, *KDR*, *EGFR*, *PIK3CA*, and *KRAS* were altered in more than two ES samples. The other genes *KIT*, *PDGFRA*, and *SRC* were mutated in one ES patient. Overall, 35% of patients in our cohort harbored one or more altered genes, thus providing a possibility for combination of gene targets for therapeutic development.

**Table 3:** Mutated genes and corresponding targeted drugs.

Gene	Type	Relevant signaling pathway	Variant frequency	Drugs
*KDR*	oncogene	PI3K-Akt	13/20	Axitinib, Regorafenib
*EGFR*	oncogene	PI3K-Akt, RTKs, Ras/Raf/MEK/ERK	3/20	Gefitinib, Lapatinib
*PIK3CA*	oncogene	PI3K-Akt	2/20	Idelalisib
*KRAS*	oncogene	PI3K-Akt, Ras family	1/20	Antroquinonol ^a^
*KIT*	oncogene	RTKs	1/20	Dasatinib
*PDGFRA*	oncogene	RTKs	1/20	Imatinib Mesylate
*SRC*	oncogene	MAPK, PI3K-Akt	1/20	Bosutinib

RTKs = receptor tyrosine kinases.

^a^ *KRAS* mutation is predictive of nonresponse to anti-EGFR therapies. The drug Antroquinonol, which directly targets KRAS, is undergoing human phase II clinical trial, and shows beneficial effects in previous anti-cancer researches [21].

## Discussion

With the ever increasing speed and efficiency, DNA sequencing has been widely used in investigating the dynamics of human genetics. In this study, 20 ES samples were assessed for mutations in oncogenes and tumor suppressor genes using the IT-PGM platform, which was launched by the Life Technologies Company in 2011. Across NGS platforms, IT-PGM displayed the fastest throughput and shortest run time (about 3 h). However, shortest reads and lowest performance with homopolymers in the IT-PGM instrument was observed [22]. Compared to traditional Sanger sequencing, IT-PGM has higher sensitivity and accuracy, above 95.0% [23]. Thus, this method was a viable platform for our study.

Patients with malignant tumors can greatly benefit from targeted therapeutic drugs if patients possess the druggable gene mutations. However, therapeutic efficacy varies from person to person greatly depending upon the biology of the tumor. Thus, there is a huge impetus to know the genetic gene profile of malignant tumors in patients so as to guide the rational design of antitumor drugs.

The *EWS-FLI1* gene fusion is the most common gene combination found in ES, and its transcript is considered as an important pathogenic factor due to its dysregulation of numerous target genes [24]. However, EWS-FLI1 fails to transform human cells in vitro, indicating that additional cooperating mutations or other factors may contribute to ES tumorigenesis [25]. TP53, IGF-1/IGF-1R, INK4A, bFGF, CD99 and relevant pathway are implicated players in ES oncogenesis [26,27]. Multi-drug chemotherapy and anti-tumor adjuvant therapies are widely used on the patients after local treatment. However, for patients with metastatic or recurrent ES, even with standard treatment, the five-year survival rates are still low. Thus, ES treatment options do not meet expectations [28]. Conversely, some antibodies such as R1507, AMG479 and tyrosine kinase inhibitors (TKIs), which mainly antagonize the insulin-like growth factor receptor-1 (IGF-1R), have played therapeutic roles in phase I and phase II clinical trials of ES [29]. Previous studies have implicated a combination of inhibitors of mTOR and IGF-1R that may improve treatment efficacy [30]. Profiling gene expression of ES is crucial in the design of personalized treatment strategies.

In the present study, 62 mutational hotspots in 26 genes across 20 ES samples were detected using IT-PGM. Variants of these hotspot genes consisted of missense and nonsense mutations, of which missense mutation occurred at higher frequencies (87.1%). Mutations in *KDR* were most prevalent, followed by *STK11*, *MLH1*, *KRAS*, *PIK3CA*, *APC*, *EGFR*, *FGFR2*, *HNF1A*, *VHL*, *IDH1*, and *PTPN11*. These genes mainly function as part of the receptor tyrosine kinase (RTK) family and the ras family. Additionally, some of these genes are implicated downstream in the phosphoinositide 3-kinase (PI3K) signaling pathway. IT-PGM evaluated these cancer-associated regions with a read depth and sensitivity that offered an unprecedented opportunity to decipher tumor heterogeneity in our ES cohort in order to glean insight about clinical therapeutic responses.

KDR, known as a type III receptor tyrosine kinase, is the main mediator of VEGF-induced endothelial activity and is considered as a significant prognostic marker in colorectal carcinoma [31]. Axitinib, a small molecule TKI targeting *KDR* and *PDGFR* mutations, was FDA-approved for renal cell carcinoma [32]. We identified the *KDR* Q472H mutation in 12 ES cases, a novel finding for ES oncogenesis. This mutation has been found in metastatic foci other than primary lesion in colorectal carcinoma and indicated as a pathogenic factor [13]. Moreover, Q472H mutation was found to increase KDR protein phosphorylation and associated with MVD in non-small cell lung cancer [33]. However, this mutation in astrocytomas showed a protective effect [34]. In ES, it has been shown that VEGF plays an important role in angiogenesis and tumorigenesis, but the mechanism involving its receptors is not clear [35,36]. More work will need to be done to understand this mutation’s specific role in ES pathogenesis.

Tumor suppressor gene *STK11* (also named *LKB1*) is located on chromosome 19p13.3, and germline mutations of it are dominant in Peutz-Jeghers Syndrome (PJS) patients [37]. Loss of function variants of this gene are also connected to sporadic and metastatic tumorigenesis such as lung adenocarcinomas, pancreatic and biliary cancers [38,39]. In our study, one D350N and three F354L amino acid changes due to missense mutations of *STK11* were firstly discovered in our ES cohort. The latter F354L mutation is thought to be deleterious in PJS patients, mainly by impairing STK11 polarizing activity and interfering with the activation of AMPK and subsequent downstream pathways [40]. Apart from it, this mutation has been described in colon cancer and serous ovarian carcinoma. Yet this mutation’s function in these cancers is unknown [41,42]. Because STK11 and PIK3CA regulate cell growth by modulating the mTOR pathway together, the PI3K/AKT pathway may be a potential target of ES [43].

Tumor supressor gene *MLH1* is essential to the mismatch repair system and associated with Lynch syndrome. Mutation of *MLH1* was found in 4 ES patients. This mutation has previously been linked to increased risk of colorectal cancer, but its significance in ES is unknown [44]. *PTPN11* encodes the non-receptor protein-tyrosine phosphatase SHP-2, which consists of two tandem Src homology 2 (SH2) domains and a PTP domain, and relays signals from activated growth factor and cytokine receptors to RAS and other signaling molecules. PTPN11 thus influences cell proliferation, differentiation, and migration. The SH2 domains are crucial in cellular signaling. Somatic gain-of-function mutations of *PTPN11* are present in certain lung adenocarcinomas, breast cancer and gastric cancer [45–47]. The A72V amino change in PTPN11 was found in one ES specimen and was located in this domain of PTPN11. Furthermore, this mutation has been seen in Juvenile Myelomonocytic Leukemia (JMML), and patients who had the mutation had poor survival outcomes. The mechanistic role of this mutation is unclear.

Mutations in the *KRAS* oncogene, particularly in codons 12 and 13, are established biomarkers for anti-EGFR therapies (cetuximab and panitumumab). In terms of our study, two ES patients harbored the *KRAS* mutations. Notably, the missense mutations leading to the G12D and G13S amino acid changes were novel findings in ES. The amino acid substitutions were along the GTP binding domain. The *KRAS* codon 12 mutation was previously reported as a negative prognostic factor for unresectable pancreatic cancer [9]. These hotspot mutations may lead to the constitutive activation of KRAS signaling pathways, which would thus activate proteins necessary for the downstream growth factors, c-Raf, and PI3K [48]. Mutations in *RAS* might mediate resistance to therapy, so the mutation state of *KRAS* needs to be assessed before using TKIs or drugs targeting *EGFR* and *PIK3CA* mutation [49].

Although several altered genes did not occur frequently in our study cohort, some of these mutations may still be of great significance to the patients. For example, somatic mutations of *EGFR* can lead to activation of the downstream signaling pathways related to cell growth and survival. In non-small cell lung cancers, these mutations are closely associated with sensitivity to TKIs. Additionally, *EGFR* mutations have been shown to play an important role in the growth of ES tumors. Inhibition of EGFR has a cytotoxic effect on ES cells in vitro, while increased EGFR expression showed resistance to the IGF-1R inhibitors [50,51]. In this study, we identified *EGFR* mutations in three patients, including two mutations, E746K and G810S, in the tyrosine kinase domain and one mutation, E114K, in the L-Domain. These are novel mutations and may be deleterious to ES patients.

IT-PGM also revealed four *APC* mutations in three cases. To note, T1292M, V1352I, and P1369S mutations were located in the mutation cluster region (MCR), a DNA region (codons 1281–1556) in which *APC* mutations cluster. These mutations have been implicated in sporadic colorectal cancers [52]. The T1292M mutation has been reported in liver cancers, and it was noted as a probable pathogenic factor listed in the ICGC database. As a key negative regulator of the Wnt pathway, APC is not only a scaffold protein but also an important protein in cytoskeleton assembly, mitotic spindle, migration, and cell-stroma interactions [53]. Mutations of *APC* have also been associated with worse outcome in urothelial carcinomas [19].

IT-PGM provided highly sensitive and quantitative mutation detection for most of the genes on the cancer panel using limited DNA quantities from our FFPE ES samples. However, there were a few difficulties in distinguishing known somatic mutations from rare germline SNPs after using the COSMIC and dbSNP databases. Other high-throughput sequencing studies showed *TP53* and *CDKN2A* mutations at different frequency in ES as compared to our data [54]. The frequency of *TP53* mutations in our samples was slightly lower (5%) compared to earlier reports which suggests that more patient samples are needed to clearly discern the *TP53* mutation pattern.

We do note that in addition to small sample size, varied findings may arise from differences in methodology and study design. It will be crucial to ascertain our collective findings in larger cohorts of patients. In this work, we also showed the necessity of using orthogonal platforms such as Sanger sequencing to validate variant calls, thereby eliminating recurrent “false positives” from further analysis.

In conclusion, we have provided a preliminary layout of the mutational landscape in ES. Such a foundation paves the groundwork for the future needed to develop personalized precise medicine, thereby not neglecting high nor low impact mutations. The high-throughput IT-PGM sequencing provided us with important information about alterations in cancer-related genes that may be critical to the dynamics of ES oncogenesis. Development of this technology may one day help address the gaps of knowledge in ES prognosis, diagnosis, and treatment.

## Supporting Information

S1 Fig Flow chart for filtering variants. It should be noted that: A) Strand-biased variants were excluded by Integrative Genomics Viewer (IGV) software and B) Variants in the AMPL339432 of *PIK3CA* was not included during our analysis as referred in the text.
(TIF)

S1 Table List of antibodies used for immunohistochemistry in the ES samples. (DOCX)

S2 Table List of 50 genes covered by Ion AmpliSeq^™^ Cancer Hotspot Panel v2. (DOCX)

S3 Table Genetic landscape of variants detected by IT-PGM. (XLSX)

S4 Table Validations of altered genes by DNA Sanger sequencing. (DOCX)

S5 Table Sequence running outputs from IT-PGM. (DOCX)
