# Correction: Molecular Heterogeneity of Ewing Sarcoma as Detected by Ion Torrent Sequencing

**DOI:** 10.1371/journal.pone.0165251

**Published:** 2016-10-18

**Authors:** Nana Zhang, Guanjun Yue, Yan Zhang, Jiangfeng You, Hua Wang

There is an error in the byline. The second author, Haijing Liu, was incorrectly added to this manuscript. Haijing Liu is not associated with this manuscript. The correct citation should read as follows: Zhang N, Yue G, Zhang Y, You J, Wang H (2016) Molecular Heterogeneity of Ewing Sarcoma as Detected by Ion Torrent Sequencing. PLoS ONE 11(4): e0153546. doi:10.1371/journal.pone.0153546 PMID: 27077911
