# Supplementary figures and images for: Molecular Heterogeneity of Ewing Sarcoma as Detected by Ion Torrent Sequencing
Source: PLoS One. 2016 Apr 14;11(4):e0153546. doi: 10.1371/journal.pone.0153546 (PMC4831808; doi:10.1371/journal.pone.0153546)

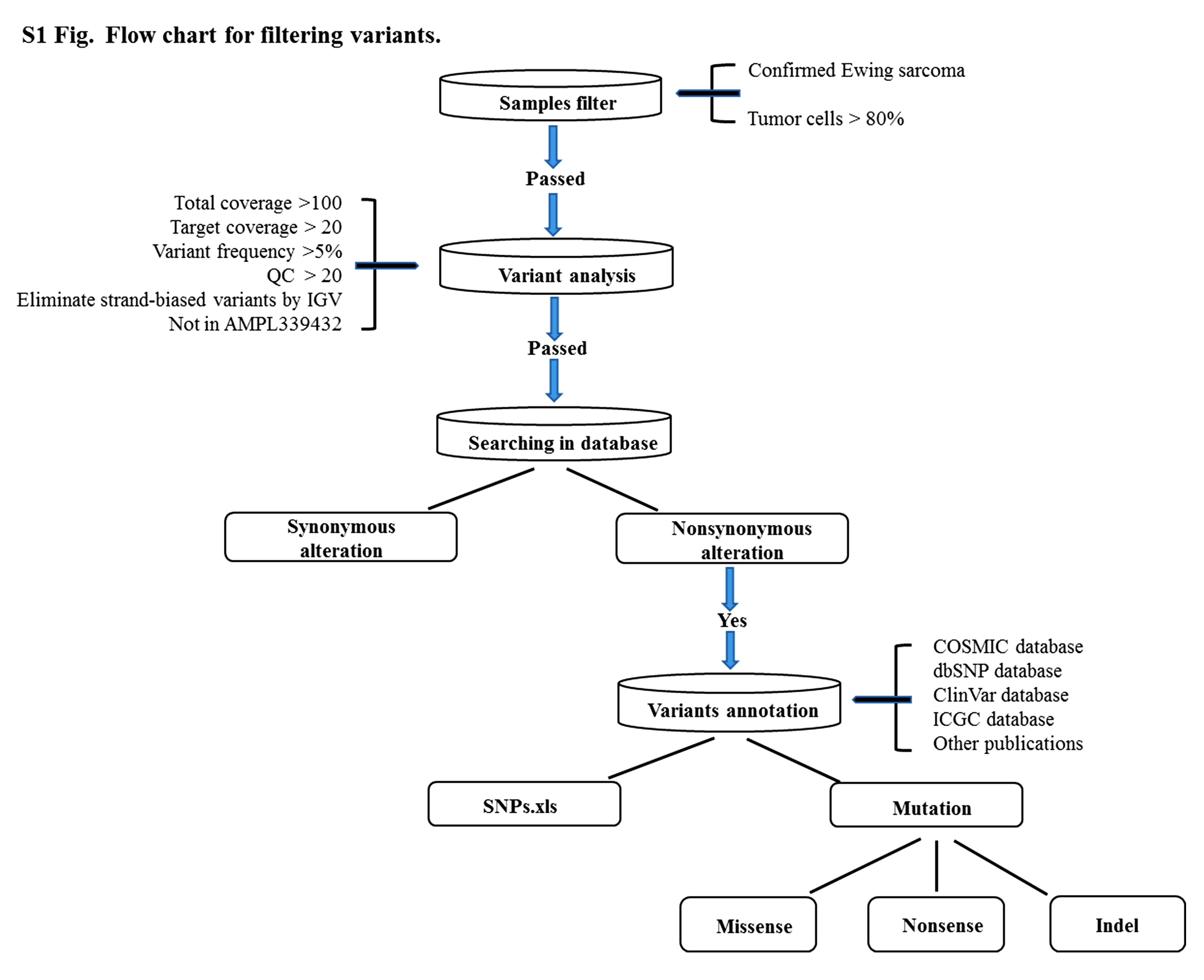

Supplement: S1 Fig — It should be noted that: A) Strand-biased variants were excluded by Integrative Genomics Viewer (IGV) software and B) Variants in the AMPL339432 of PIK3CA was not included during our analysis as referred in the text. (TIF) [file pone.0153546.s001.tif]
