# Supplementary material for: Molecular Heterogeneity of Ewing Sarcoma as Detected by Ion Torrent Sequencing
Source: PLoS One. 2016 Apr 14;11(4):e0153546. doi: 10.1371/journal.pone.0153546 (PMC4831808; doi:10.1371/journal.pone.0153546)
Supplement: S1 Table — (DOCX) [file pone.0153546.s002.docx]

**S1 Table. List of antibodies used for immunohistochemistry in the ES samples.**

|  | | | | |  |
| --- | --- | --- | --- | --- | --- |
| **Antibody** | | **Species** | **Clone** | **Dilution** | **Source** |
| CD99 | | Mouse monoclonal | 013 | 1:60 | Dako, Glostrup, Denmark |
| FLI1 | | Mouse monoclonal | MRQ-1 | Ready-to-use | ZETA,California, USA. |
| Ki-67 | | Mouse monoclonal | MIB-1 | 1:100 | Dako, Glostrup, Denmark |
| Syn | | Rabbit polyclonal | EP158 | 1:200 | EPITMICS, California, USA |
| CgA | | Rabbit polyclonal | DAK-A3 | 1:800 | Dako, Glostrup, Denmark |
| NSE | | Mouse monoclonal | NSE-P1+NSE-P2 | Ready-to-use | ZETA,California, USA. |
| S-100 | | Rabbit polyclonal | poly | 1:100 | Dako, Glostrup, Denmark |
