# Supplementary material for: Molecular Heterogeneity of Ewing Sarcoma as Detected by Ion Torrent Sequencing
Source: PLoS One. 2016 Apr 14;11(4):e0153546. doi: 10.1371/journal.pone.0153546 (PMC4831808; doi:10.1371/journal.pone.0153546)
Supplement: S2 Table — (DOCX) [file pone.0153546.s003.docx]

| **S2 Table. List of 50 genes covered by** **Ion AmpliSeq™ Cancer Hotspot Panel v2** | | | | |
| --- | --- | --- | --- | --- |
| *ABL1* | *AKT1* | *ALK* | *APC* | *ATM* |
| *BRAF* | *CDH1* | *CDKN2A* | *CSF1R* | *CTNNB1* |
| *EGFR* | *ERBB2* | *ERBB4* | *EZH2* | *FBXW7* |
| *FGFR1* | *FGFR2* | *FGFR3* | *FLT3* | *GNA11* |
| *GNAS* | *GNAQ* | *HNF1A* | *HRAS* | *IDH1* |
| *JAK2* | *JAK3* | *IDH2* | *KDR* | *KIT* |
| *KRAS* | *MET* | *MLH1* | *MPL* | *NOTCH1* |
| *NPM1* | *NRAS* | *PDGFRA* | *PIK3CA* | *PTEN* |
| *PTPN11* | *RB1* | *RET* | *SMAD4* | *SMARCB1* |
| *SMO* | *SRC* | *STK11* | *TP53* | *VHL* |
