# Supplementary material for: Molecular Heterogeneity of Ewing Sarcoma as Detected by Ion Torrent Sequencing
Source: PLoS One. 2016 Apr 14;11(4):e0153546. doi: 10.1371/journal.pone.0153546 (PMC4831808; doi:10.1371/journal.pone.0153546)
Supplement: S4 Table — (DOCX) [file pone.0153546.s005.docx]

| **S4 Table. Validations of altered genes by DNA Sanger sequencing.** | | | |  |  |  |  |
| --- | --- | --- | --- | --- | --- | --- | --- |
| **Samples** | **COSMIC ID/**  **dbSNP ID** | **Gene** | **Mutation CDS** | **Mutation AA** | **Variant  Frequency (%)** | **Variant Type** | **Verification  Result** |
| T13 | COSM149673 | *KDR* | c.1416A>T | p.Gln472His | 100.00 | missense | yes |
| T19 | COSM26085 | *MLH1* | c.1151T>A | p.Val384Asp | 49.55 | missense | yes |
| T1 | COSM21360 | *STK11* | c.1062C>G | p.Phe354Leu | 54.91 | missense | yes |
| T8 | COSM521 | *KRAS* | c.35G>A | p.Gly12Asp | 24.33 | missense | yes |
| T8 | COSM 13015 | *PTPN11* | c.215C>T | p.Ala72Val | 28.05 | missense | yes |
| T13 | rs1042522 | *TP53* | c.98C>G | p.Pro33Arg | 52.94 | missense | yes |
| T14 | --- | *TP53* | c.707C>T | p.Cys236Tyr | 53.90 | missense | yes |
| T18 | rs41115 | *APC* | c.4479G>A | p.(=) | 100.00 | synonymous | yes |
| T14 | rs1050171 | *EGFR* | c.2361G>A | p.(=) | 50.21 | synonymous | yes |
| T19 | --- | *PDGFRA* | c.1701A>G | p.(=) | 100.00 | synonymous | yes |
| T15 | rs1800861 | *RET* | c.2307G>T | p.(=) | 64.32 | synonymous | yes |
| T14 | rs1800863 | *RET* | c.2712C>G | p.(=) | 50.66 | synonymous | yes |

‘---’ refers to no record in the COSMIC or dbSNP database.


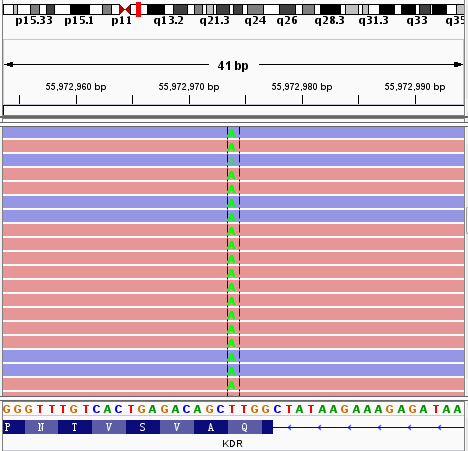


**T13** ***KDR*: chr4:55972974 T>A**


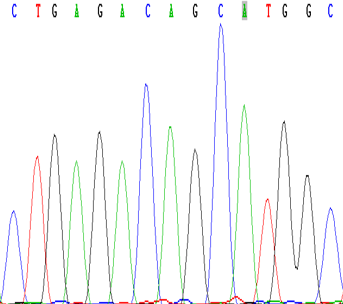


Ion Torrent

Sanger

**Ion Torrent and Sanger Sequencing verification results**

Ion Torrent

Sanger

**T19 *MLH1*: chr3:37067240 T>A**


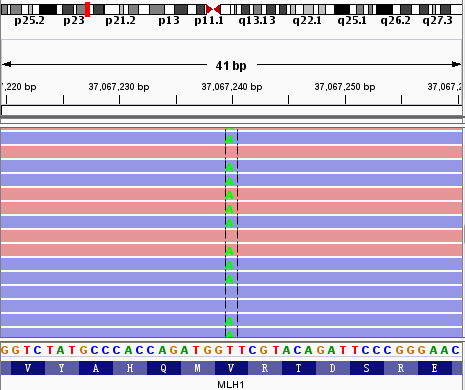

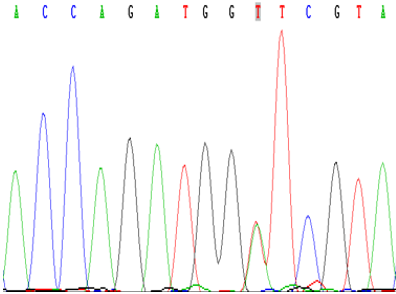


Ion Torrent


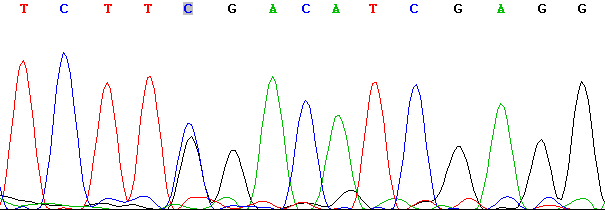

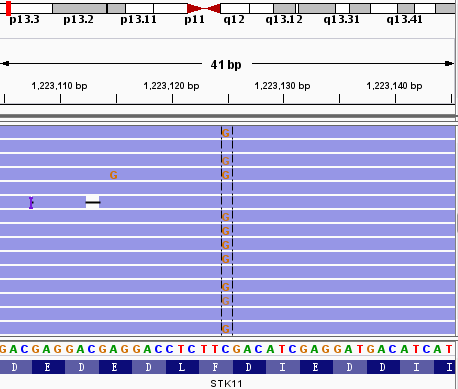


**T1** ***STK11*: chr19:1223125 C>G**

Sanger

**T8** ***KRAS:* chr12:25398284 C>T**


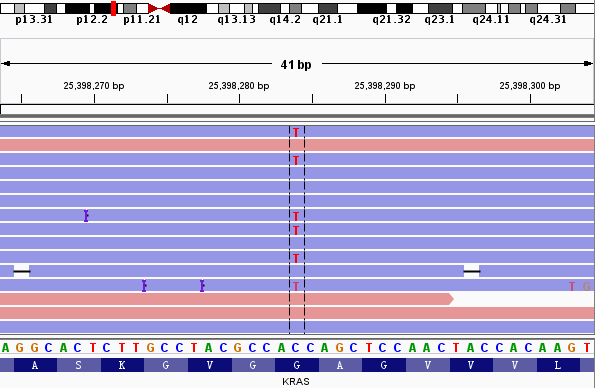


Ion Torrent

Sanger


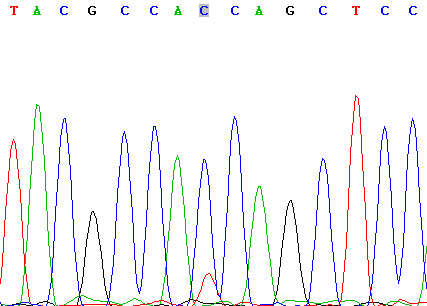


**T8 *PTPN11*: chr12:112888199 C>T**


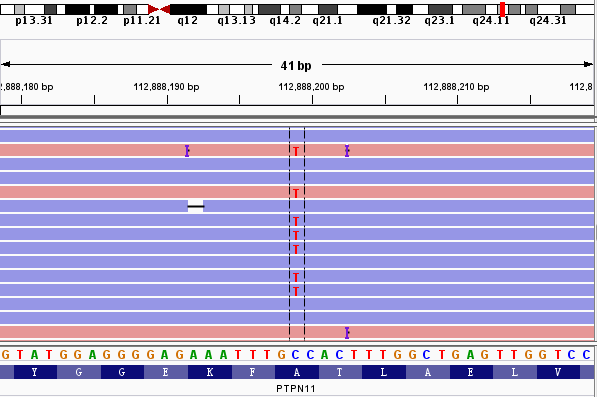

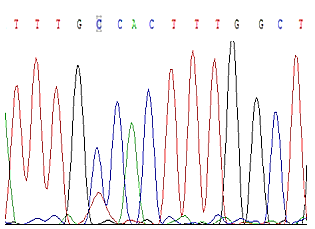


Ion Torrent

Sanger


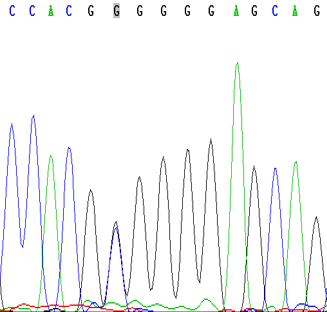


**T13 *TP53*: Chr17:7579472 G>C**


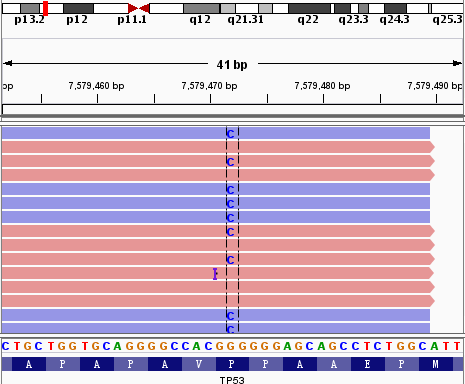


Ion Torrent

Sanger


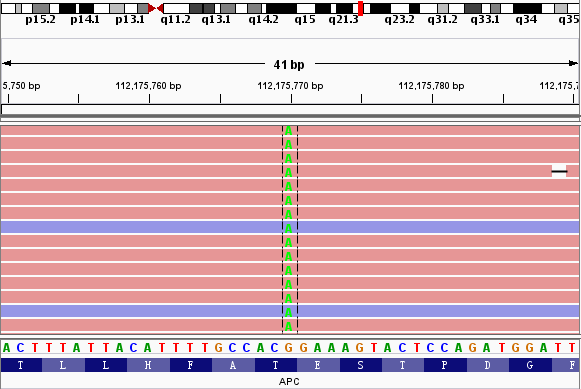


**T18** ***APC*: chr5:** **112175770 G>A**


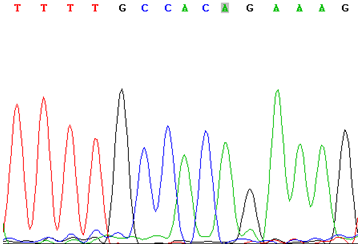


Ion Torrent

Sanger


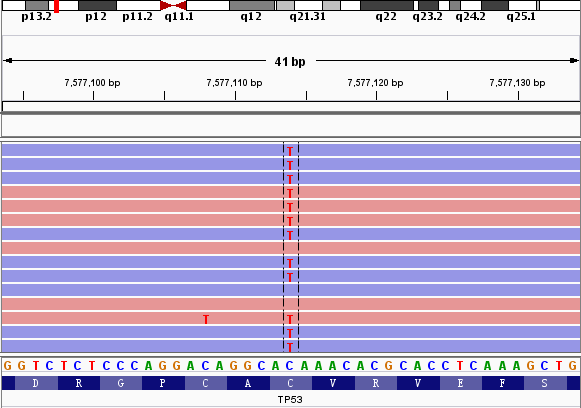


**T14 *TP53*: chr17:** **7577114 C>T**


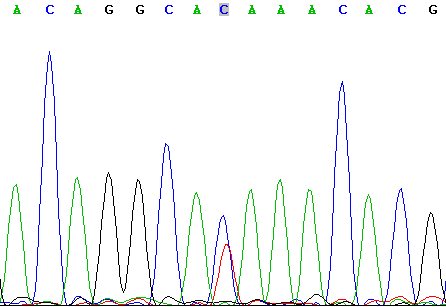


Ion Torrent

Sanger

Ion Torrent

Sanger

**T14** ***EGFR*: chr7:55249063 G>A**


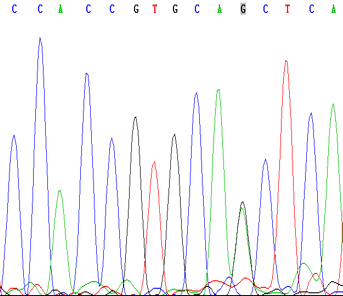

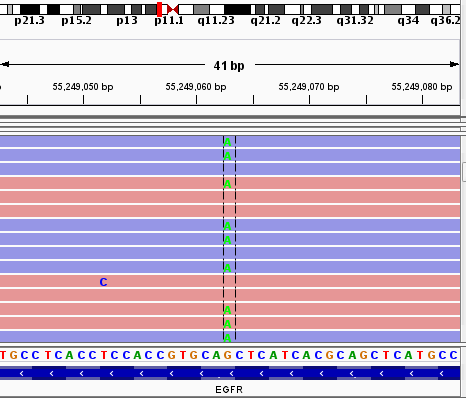


Ion Torrent


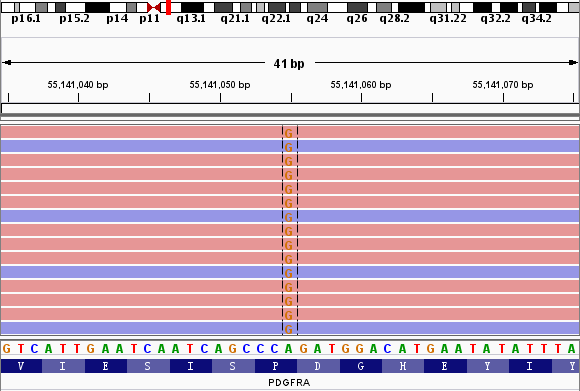


**T19** ***PDGFRA*: chr4:** **55141055 A>G**


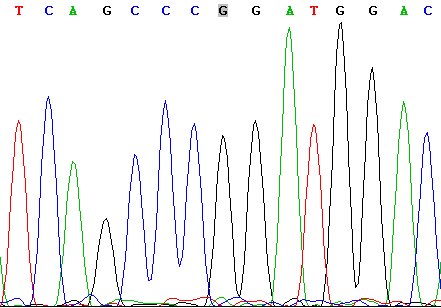


Sanger

Sanger

Ion Torrent


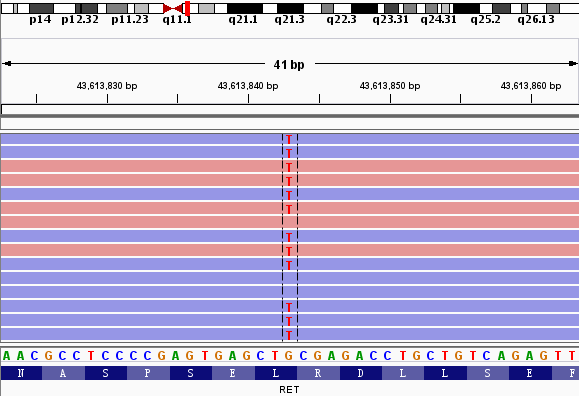


**T15** ***RET*: chr10:** **43613843 G>T**


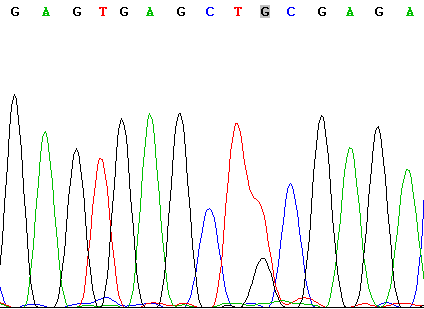


Ion Torrent


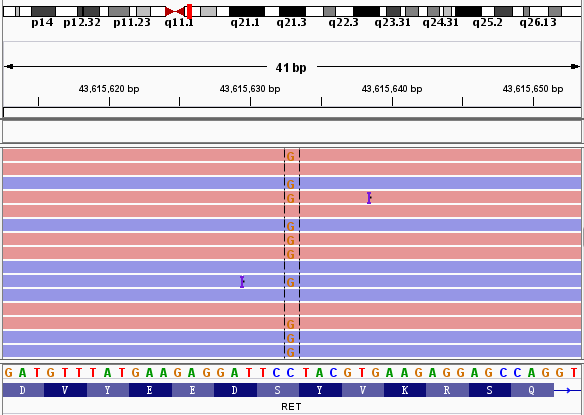


**T14** ***RET*: chr10:** **43615633 C>G**


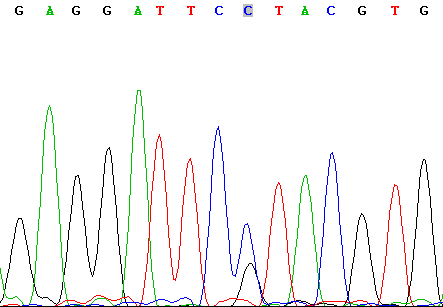


Sanger

**Sanger sequencing for *KDR* Q472H mutations in other 11 cases**


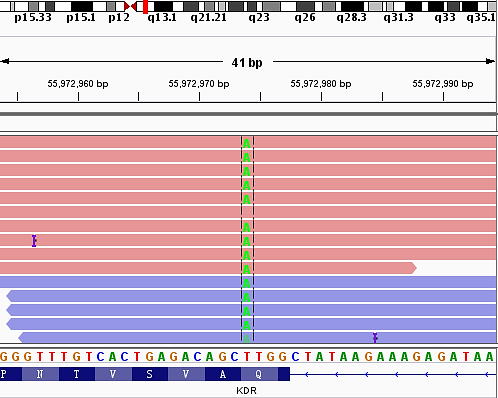


**T7 *KDR*: chr4:55972974 T>A**


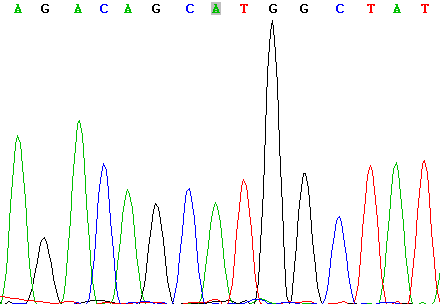


Ion Torrent

Sanger


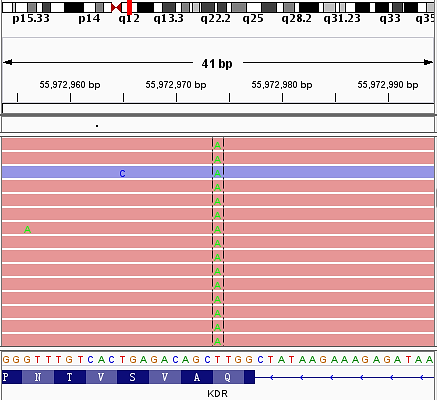


**T8 *KDR*: chr4:55972974 T>A**


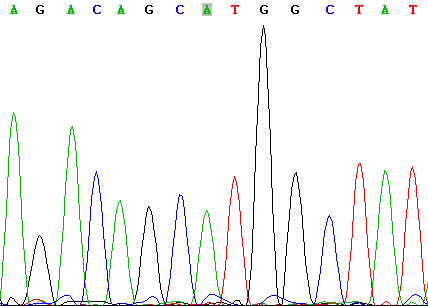


Ion Torrent

Sanger


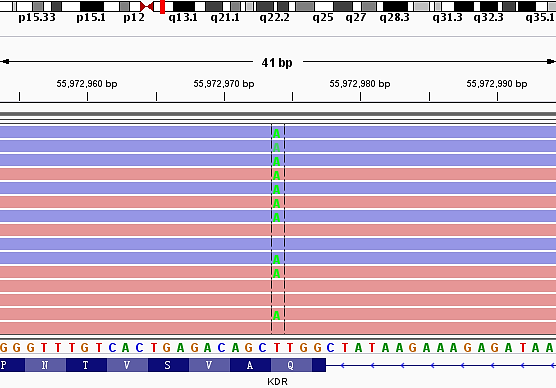


**T9 *KDR*: chr4:55972974 T>A**


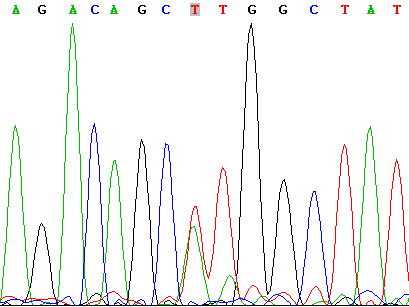


Ion Torrent

Sanger


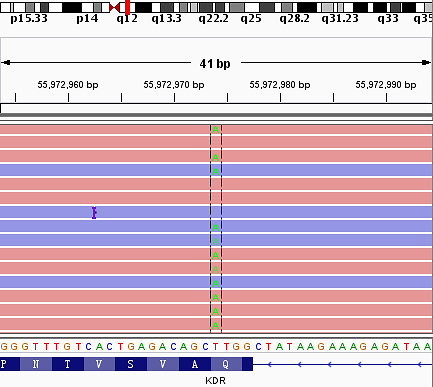


**T10 *KDR*: chr4:55972974 T>A**


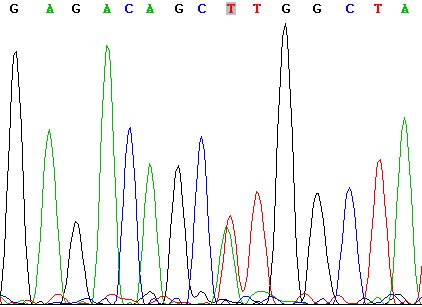


Ion Torrent

Sanger


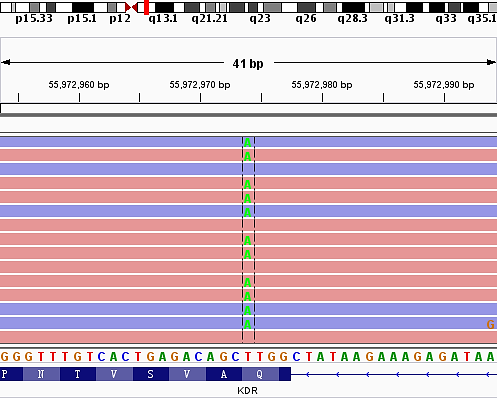


**T11 *KDR*: chr4:55972974 T>A**


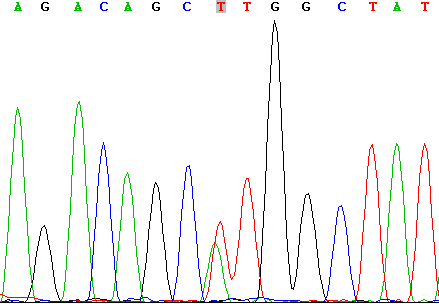


Ion Torrent

Sanger


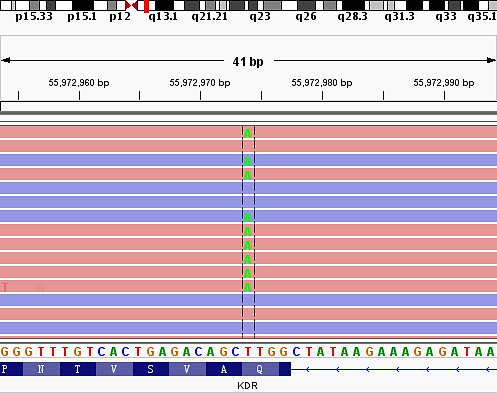


**T12 *KDR*: chr4:55972974 T>A**


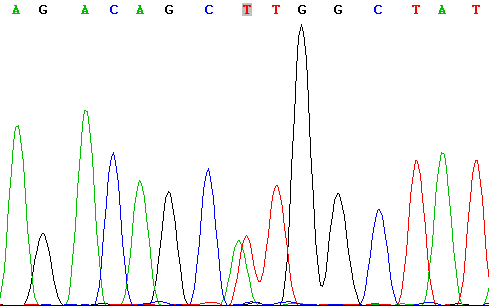


Ion Torrent

Sanger


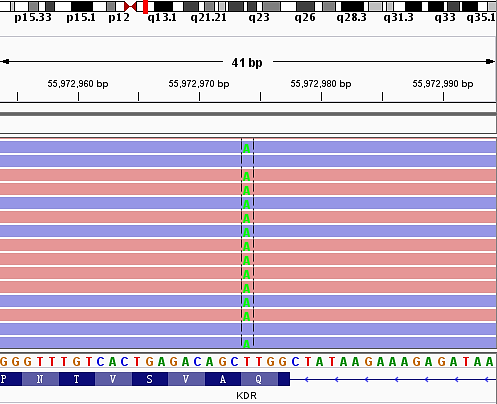


**T15 *KDR*: chr4:55972974 T>A**


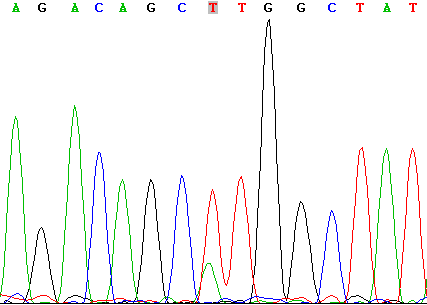


Ion Torrent

Sanger

0


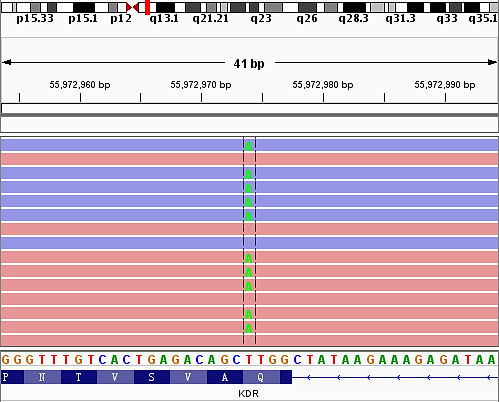


**T16 *KDR*: chr4:55972974 T>A**


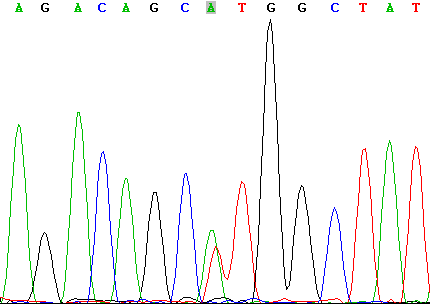


Ion Torrent

Sanger


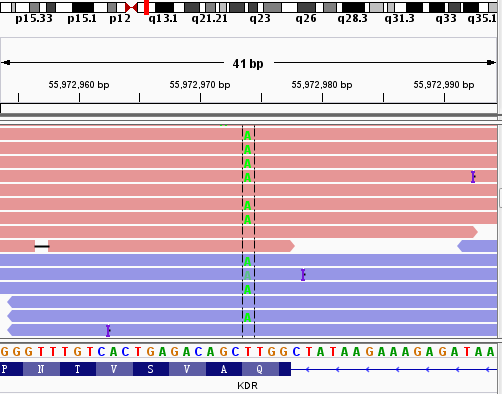


**T17 *KDR*: chr4:55972974 T>A**


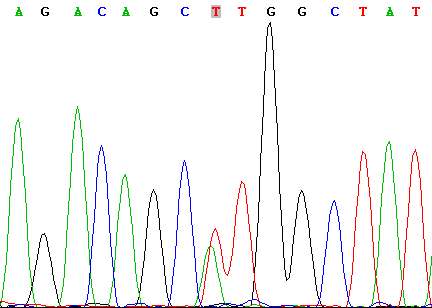


Ion Torrent

Sanger

**T18 *KDR*: chr4:55972974 T>A**


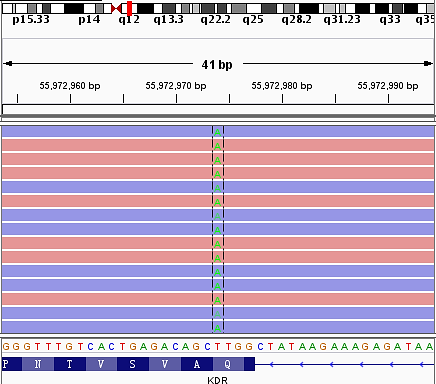

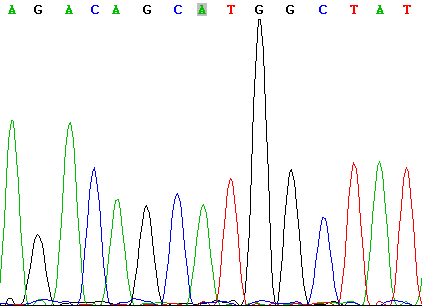


Ion Torrent

Sanger


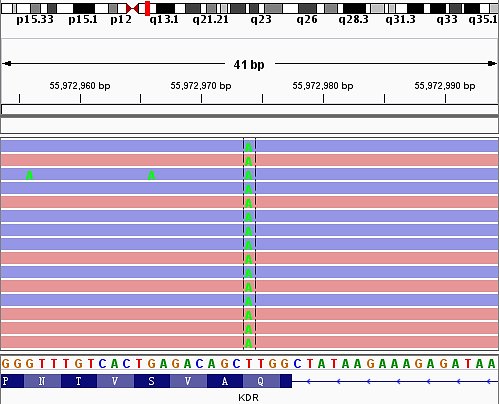


**T20 *KDR*: chr4:55972974 T>A**


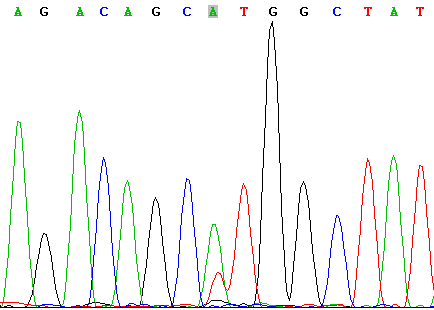


Ion Torrent

Sanger
