# Supplementary material for: Molecular Heterogeneity of Ewing Sarcoma as Detected by Ion Torrent Sequencing
Source: PLoS One. 2016 Apr 14;11(4):e0153546. doi: 10.1371/journal.pone.0153546 (PMC4831808; doi:10.1371/journal.pone.0153546)
Supplement: S5 Table — (DOCX) [file pone.0153546.s006.docx]

| **S5 Table. Sequencing outputs from IT-PGM** | | | |
| --- | --- | --- | --- |
| **Samples** | **Total Reads** | **Mean Read**  **Length (bp)** | **Average reads per amplicon** |
| T1 | 372765 | 95 | 1736 |
| T2 | 349852 | 100 | 1590 |
| T3 | 297707 | 91 | 1266 |
| T4 | 271994 | 105 | 1266 |
| T5 | 426698 | 93 | 2003 |
| T6 | 435738 | 108 | 2071 |
| T7 | 329513 | 90 | 1522 |
| T8 | 273908 | 96 | 1257 |
| T9 | 427743 | 108 | 2031 |
| T10 | 541456 | 108 | 2549 |
| T11 | 451192 | 109 | 2055 |
| T12 | 414486 | 108 | 1929 |
| T13 | 446652 | 119 | 2081 |
| T14 | 421527 | 121 | 1912 |
| T15 | 464340 | 118 | 2163 |
| T16 | 417947 | 112 | 1952 |
| T17 | 383348 | 98 | 1779 |
| T18 | 561600 | 108 | 2676 |
| T19 | 443279 | 119 | 2058 |
| T20 | 405585 | 107 | 1863 |
